# Do LRG1–SERPINA1 Interactions Modulate Fibrotic and Inflammatory Signatures in Rheumatoid Arthritis? A Proteomic and In Silico Investigation

**DOI:** 10.3390/pathophysiology33010016

**Published:** 2026-02-06

**Authors:** Talib Hussain, Monika Verma, Sagarika Biswas

**Affiliations:** 1 Integrative and Functional Biology Department, Council of Scientific & Industrial Research (CSIR)-Institute of Genomics and Integrative Biology, Mall Road, Delhi University Campus, Delhi 110007, India; talibhu23@gmail.com (T.H.); vmonika973@gmail.com (M.V.); 2 Academy of Scientific and Innovative Research (AcSIR), Ghaziabad 201002, India

**Keywords:** Rheumatoid arthritis, inflammation, fibrosis, LRG1, molecular dynamics

## Abstract

**Background:** Rheumatoid arthritis (RA) is a systemic, pro-inflammatory, autoimmune disease that mainly affects the joints in a symmetrical manner. Differential proteomic profiling through Sequential Window Acquisition of all Theoretical Fragment Ion Mass Spectra (SWATH-MS/MS) helps in a better understanding of the RA pathogenesis. In this study, we compared the differentially upregulated proteins with those associated with fibrosis to gain a deeper understanding of the fibrotic aspect of RA. **Methods:** We analyzed plasma proteomics data, previously obtained by SWATH-MS/MS. Our focus was on proteins associated with Leucine Rich Alpha2glycoprotein1 (LRG1) and we employed an in silico method. **Results:** We identified common proteins between RA and fibrosis. Among them, LRG1 and Serine Protease Inhibitor Clade A, Member 1 (SERPINA1) showed a high co-expression score in the gene clusters. LRG1 is both pro-inflammatory and pro-fibrotic, while SERPINA1 is an anti-inflammatory protein that inhibits pro-inflammatory and pro-fibrotic molecules (Elastase). Further, docking studies and a simulation study of the docked complexes with the analysis of Hydrogen bonds, Solvent Accessible Surface Area (SASA), Root Mean Square Deviation (RMSD), Root Mean Square Fluctuation (RMSF) and Radius of gyration (Rg), suggested a strong interaction between the two partners, LRG1 and SERPINA1. **Conclusions**: Our study suggests that LRG1 may inhibit SERPINA1 and promote inflammation and fibrotic processes by disrupting SERPINA1’s primary function.

## 1. Introduction

Rheumatoid arthritis (RA) is a chronic autoimmune disorder that mainly affects the synovial joints. It causes ongoing inflammation, pain, and tissue damage, affecting about 1% of the global population, and is more common in women [1,2]. The disease typically begins with inflammation of the synovial membrane, resulting in swelling, warmth, and stiffness, particularly in the joints of the hands and feet. Over time, ongoing inflammation damages the articular cartilage and the underlying bone, resulting in joint deformity [3]. The ongoing inflammation in RA changes normal synovial tissue into an aggressive, growing pannus that invades cartilage and bone [4]. Immune mediators, such as tumor necrosis factor-alpha (TNF-α), interleukin-6 (IL-6), and IL-17, drive this destructive process [5]. Rheumatoid Arthritis Fibroblast-like Synoviocytes (RA-FLS) and macrophages also play important roles in contributing to tissue damage by releasing degradative enzymes and promoting the formation of new blood vessels in the joints [6,7,8]. RA-FLS have drawn attention in RA pathogenesis due to their dual role in the progression of RA. Initially, they respond to inflammation by releasing cytokines and enzymes that break down the matrix, such as matrix metalloproteinases (MMPs) [6]. However, continued stimulation and long-term disease effects cause them to become abnormally active, leading to a tumour-like behavior [9]. These overactive RA-FLS produce excessive extracellular matrix (ECM), which promotes scarring in the synovial tissue [10]. The fibrosis in RA is not solely the result of inflammation, but it is a distinct pathological process that involves Transforming Growth Factor Beta (TGF-β) signaling, alterations in ECM turnover, and the transformation of cells into myofibroblasts [11,12]. Recent research indicates that although anti-inflammatory drugs, such as TNF-α and IL-6 inhibitors, can effectively reduce inflammation, they often fail to prevent fibrotic changes and show significant side effects when used for prolonged periods [13,14,15]. This suggests that inflammation and fibrosis, though related, operate as partially independent processes in RA. Further, the literature reports show that fibrotic markers such as Fibronectin1 (FN1), Alpha Smooth Muscle Actin (α-SMA), and TGF-β are highly expressed in RA-FLS [11,16]. The TGF-β pathway has also been found to be highly active in RA [11,17].

We, therefore, explored the inflammatory and fibrotic properties of proteins that contribute to RA pathogenesis. Differentially expressed proteins play a significantly important role in the investigation process. To identify proteins of interest, we analyzed differential proteins in RA plasma by Sequential Window Acquisition of all Theoretical Fragment Ion Mass Spectra (SWATH-MS/MS) and identified proteins that were significantly differentially expressed in RA [18]. We compared the up-regulated proteins of RA plasma with RA-associated genes from DisGeNET and fibrosis-associated genes from the Gene Card database. A possible interaction between Leucine Rich Alpha2glycoprotein1 (LRG1) and SERine Protease INhibitor, Clade A, Member 1 (SERPINA1) was thus revealed. Further interactions were studied through docking and simulation. Our study suggests that LRG1 may alter the protective function of SERPINA1 by binding to its functionally active sites. LRG1 expression is significantly upregulated in RA plasma [19], and its knockout ameliorated arthritis in mice and reduced Th17 differentiation [20]. The results thus obtained in our study, together with literature support, suggest that LRG1 can be considered as a potential therapeutic target in RA pathogenesis.

## 2. Methodology

### 2.1. Proteomic Data Analysis

The previously obtained SWATH-MS proteomic data from 60 plasma samples of RA patients and 40 from healthy individuals, collected at All India Institute of Medical Sciences (AIIMS), Delhi, India, were analyzed. Proteins that were significantly upregulated, with a threshold of at least 1.6-fold increase relative to healthy controls (HC), were considered. Proteins that met this criterion were selected, and gene symbols were used for further examination [18].

### 2.2. Common Gene Identification

To identify genes associated with RA and fibrosis, two datasets were sourced from public databases: RA-related genes from DisGeNET (v25.4) [21] and fibrosis-related genes from GeneCards (v5.26) [22]. All gene sets were standardized to a common set of gene symbols and matched with SWATH-upregulated genes.Venny (v2.1.0) was used to identify overlaps among the three datasets [1].

### 2.3. Protein–Protein Interaction (PPI) and Functional Enrichment Analysis

The overlapping genes were analyzed for PPIs using the STRING (v12.0) database. The interaction network was then exported and visualized using Cytoscape (v3.10.3). Functional enrichment analysis was performed in Cytoscape to identify enriched Reactome pathways, Biological Processes (BP), Cellular Components (CC), Molecular Functions (MF), and Tissue Expression patterns related to the common proteins. Graphs were generated using RStudio (v 2025.09.1+401) software [23,24,25].

### 2.4. Target Protein Selection and Molecular Docking

Using RStudio, Principal Component Analysis (PCA) was performed on the three gene sets, based on Fold Change (FC), Gene Disease Association (GDA), and Relevance scores. The 3D structures of proteins were obtained from the Research Collaboratory for Structural Bioinformatics Protein Data Bank (RCSB-PDB). Additionally, the co-expression score of the closely clustered genes of LRG1 was considered, along with protein–protein docking scores, as a part of the selection criteria. Docking scores were obtained by using ClusPro (v2.0) [23] and PyMOL (v3.1.6.1) [26] for visualization. LigPlus (v2.2.9) software was used to analyze hydrogen bond formation between proteins [21,27]. The total energy calculation by Molecular Mechanics/Generalized Born Surface Area (MMGBSA) was carried out using the Hawkdock (v2) server [26].

### 2.5. Molecular Dynamics Simulation (MD)

To evaluate the structural stability and dynamic behavior of the docked complexes, we performed MD simulations with GROMACS 2024.2. Each complex underwent a 25-nanosecond (ns) simulation utilizing the CHARMM27 all-atom force field and the TIP3P explicit water model [27,28,29]. The complexes were placed in a cubic simulation box, ensuring a uniform distance of 1.0 nm between the solute and the box edges. To achieve a physiological ionic strength, Na^+^ and Cl^−^ ions were added to attain a concentration of 0.15 M, which helped to maintain a neutral system. For the initial relaxation of each complex, we used the steepest descent algorithm to minimize energy until the forces dropped to below 1.0 kcal/mol. All simulations were performed under isothermal-isobaric (NPT) conditions, maintaining the number of particles, pressure, and temperature constant. The temperature was maintained at 300 K and the pressure at 1 atm. We used periodic boundary conditions in all directions to simulate an infinite system and to minimize edge effects. The trajectories from the simulations were analyzed to evaluate key structural and dynamic parameters, including root-mean-square deviation (RMSD), root-mean-square fluctuation (RMSF), radius of gyration (Rg), solvent-accessible surface area (SASA), and intermolecular hydrogen bonding. The average values from the 25 ns trajectories were compared across all complexes to highlight differences in their conformational stability and compactness [30].

## 3. Results

### 3.1. Identification of Common Proteins and PCA Analysis

**Figure 1:**
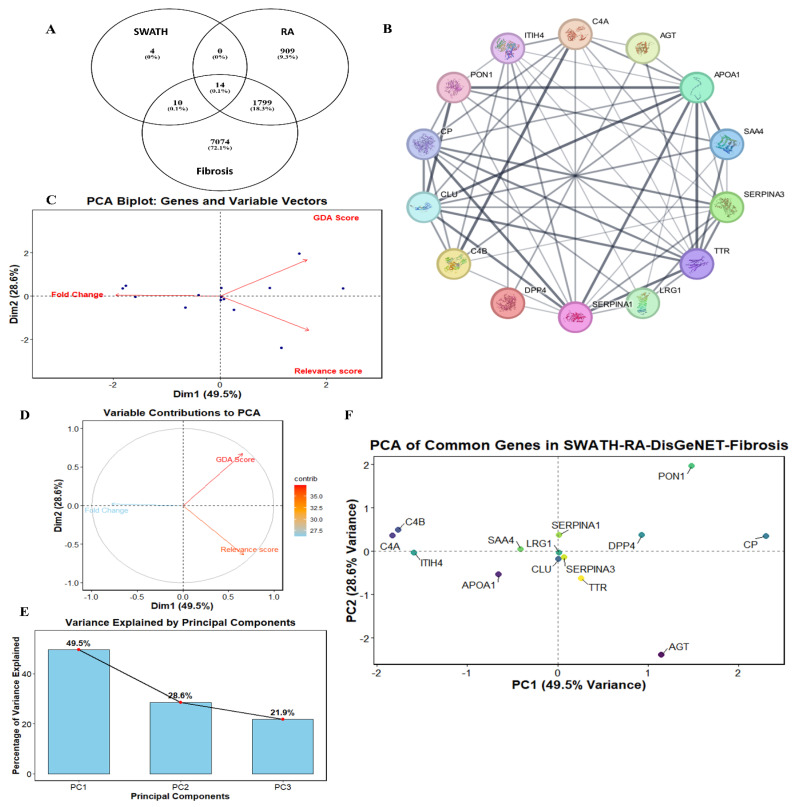
Protein–Protein network analysis overlapping with RA and fibrosis. Venn diagram showing 14 common proteins between SWATH, RA, and fibrosis (**A**) and common interacting proteins (**B**). PCA analysis showing the correlations among FC, GDA score, and relevance score for the 14 common gene sets. GDA shows a negative correlation with FC and Relevance score, while FC and Relevance score are positively correlated (**C**). GDA score contributes more to the overall variation among the variables shown in the unit circle (**D**). Bar plot showing the variations among the variables with 49.5%, 28.6%, and 21.9% in PC1, PC2, and PC3, respectively (**E**). Gene distribution chart showing the cluster of LRG1, SERPINA1, SERPINA3, and Clu around the center, suggesting a close association among the variables (**F**).

The SWATH-MS analysis identified 28 proteins that were significantly upregulated (≥1.6-fold) (Supplementary Table S1). When these proteins were intersected with RA genes from the DisGeNET database (Supplementary Table S2) and fibrosis genes from the GeneCards database (Supplementary Table S3), 14 common genes were identified in both inflammatory and fibrotic processes in RA (Figure 1A). Figure 1B shows the interaction network of the 14 common proteins analyzed in Cytoscape. The PCA analysis revealed that the FC is negatively correlated with the GDA and Relevance score variables, since it is directed in the opposite direction of those variables. However, since the GDA and Relevance variables are in the same direction, they are positively correlated (Figure 1C). This showed that genes with high FC expression are less associated with respect to RA. Figure 1D showed that the GDA score contributes more to PCA than the other two variables (FC and Relevance score). The FC vector that appears longer in the PCA bi-plot showed a stronger correlation with the principal components (Figure 1C). It is valuable to consider the GDA variable, which contributed even more to the explained variance, indicated by its higher contribution in the unit circle (Figure 1D). These refinements highlighted that FC is effectively represented by the correlation in the PC1–PC2 projection, while GDA showed the overall variance across the PCs. The bar plot for the same has been plotted to explain and visualize the variance, which showed 49.5%, 28.6% and 21.9% variance in PC1, PC2, and PC3, respectively (Figure 1E). The distribution of the genes in the PCA plot showed that LRG1, SERPINA1, SERPINA3, and CLU form a close cluster (Figure 1F). The clustering around the center of the plot is due to low correlation among the proteins for FC, GDA, and Relevance score, further supported by low co-expression scores from STRING (Supplementary Table S4). Since low co-expression scores do not rule out the possibility of interactions within the cluster, we also sorted them by docking scores and hydrogen-bond formation. The clustering does not indicate a direct physical interaction. Rather, it reflects an average association with inflammation and fibrosis, as evidenced by the moderate FC, GDA, and Relevance Scores. Further confirmation was therefore attempted by investigating their possible interactions using molecular docking.

### 3.2. Co-Expression and Functional Enrichment Analysis

**Figure 2:**
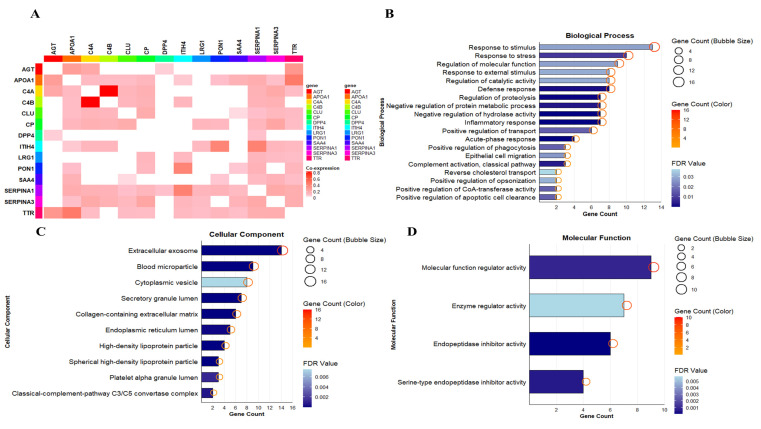

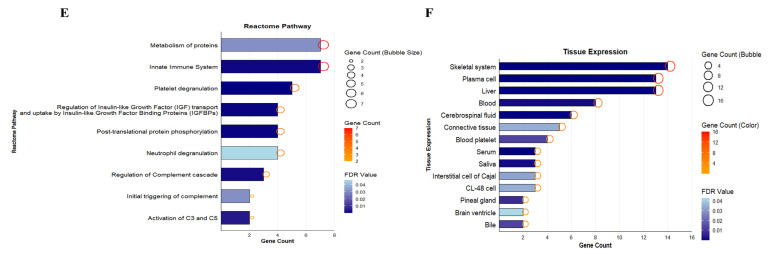
Functional enrichment analysis of common proteins overlapping with RA and fibrosis. Functional enrichment analysis of the 14 common interaction proteins. The sizes of the circles and the colors in the figures represent the number of genes, and the colors of the bars represent the false discovery rate (FDR) values. Heat map analysis of co-expressing partners among the common 14 interacting proteins (**A**). LRG1 has a co-expression score of 0.133, 0.102 with SERPINA1 and SERPINA3, respectively. LRG1 does not show any co-expression with Clu. LRG1 is involved in cell migration, responding to stimuli, and stress in the biological process (**B**). In the cellular component, LRG1 occurs in extracellular exosomes (**C**), and in Molecular Function, SERPINA1, the co-expressing partner of LRG1, is involved in endopeptidase inhibitory activities (**D**). The reactome pathway illustrates the role of LRG1 in neutrophil degranulation and immune responses (**E**). Tissue expression analysis showed that liver, saliva, cerebrospinal fluid, skeletal system, and plasma cells contribute to LRG1 expression, showing its wide expression (**F**).

The PPI network generated in STRING showed strong connectivity among the 14 proteins, suggesting functional relationships. However, since LRG1, SERPINA1, SERPINA3, and Clusterin (CLU) form a close cluster, SERPINA1, SERPINA3, and CLU exhibit anti-inflammatory properties [31,32,33]. LRG1 specifically promotes inflammation in CIA mice [20], fibrosis in Osteoarthritis fibroblast-like synoviocytes (OA-FLS) [34], and renal fibrosis [35]. These activities thus made LRG1 a suitable candidate for interaction studies with the closely clustered anti-inflammatory proteins (SERPINA1, SERPINA3, and CLU). LRG1 was found to have a co-expression score of 0.133 and 0.102 with SERPINA1 and SERPINA3, respectively (Figure 2A) (Supplementary Table S4). However, LRG1 did not show any co-expression with CLU. Although LRG1 and SERPINA1 have a very low co-expression score, proteins with low co-expression can still engage in significant interactions [36]. Since the co-expression score is very low across all clustered pairs, we further analyzed their docking energies to further evaluate our selection.

The enrichment analysis conducted in Cytoscape highlighted several important biological categories (Supplementary Tables S5–S9). In the Biological Process, LRG1 was found to be involved in cell migration, stimulus response, and stress. However, SERPINA1 was found to be involved in Response to stimulus, Response to stress, Regulation of molecular function, Defense response, Regulation of catalytic activity, Negative regulation of hydrolase activity, Inflammatory response, Regulation of proteolysis, Negative regulation of protein metabolic process, and Acute-phase response (Figure 2B). These findings showed that both LRG1 and SERPINA1 are involved in stimulus and stress responses. In the Cellular Component, LRG1 was found to occur in the extracellular exosomes and SERPINA1 in the Extracellular exosome, Cytoplasmic vesicle, Secretory granule lumen, Collagen-containing extracellular matrix, Endoplasmic Reticulum lumen, and Platelet alpha granule lumen (Figure 2C). The results obtained thus revealed the occurrence of LRG1 and SERPINA1 in the extracellular components. In Molecular Function, SERPINA1 was found to be involved in endopeptidase inhibition. This showed that SERPINA1 is involved in protease-inhibitory activity (Figure 2D). The Reactome Pathway showed that LRG1 is involved in neutrophil degranulation and immune responses. SERPINA1 in Innate Immune System, Metabolism of proteins, Platelet degranulation, Post-translational protein phosphorylation, Regulation of Insulin-like Growth Factor (IGF) transport and uptake by Insulin-like Growth Factor Binding Proteins (IGFBPs), and Neutrophil degranulation. These findings showed that LRG1 and SERPINA1 is involved in the immune related pathways (Figure 2E). Tissue expression analysis showed the involvement of liver, saliva, cerebrospinal fluid, skeletal system, and plasma cells in LRG1 expression, whereas SERPINA1’s expression was found in plasma cells, skeletal system, cerebrospinal fluid, liver, blood, bile, blood platelets, interstitial cells of Cajal, connective tissue, and CL-48 cells. These showed a wide range of expressions (Figure 2F). Thus, in a nutshell, the enrichment analysis revealed the involvement of LRG1 and SERPINA1 in inflammation and immune-related functions.

### 3.3. Protein–Protein Docking Analysis

**Figure 3:**
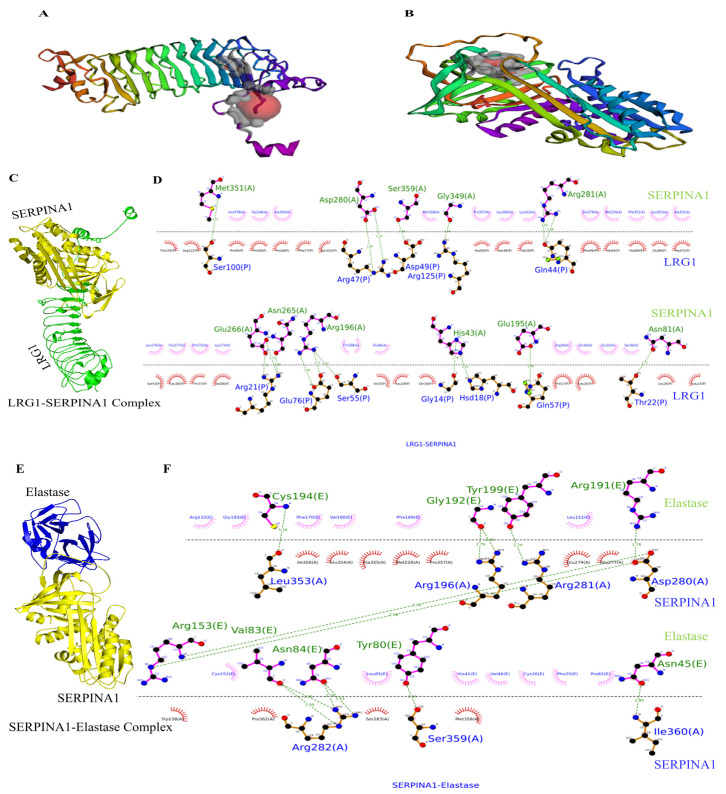
Molecular docking analysis of protein–protein complexes. Crystal structure of LRG1 and SERPINA1 with their active pocket sites (**A**,**B**), respectively. LRG1–SERPINA1 docked complex structure (**C**) and its interaction and hydrogen bond formation, with a total of 17 hydrogen bonds, suggesting LRG1’s efficient binding capability with SERPINA1 (**D**). SERPINA1-Elastase docked complex structure (**E**) and its interaction with hydrogen bond formation, with a total of 13 hydrogen bonds (**F**).

**Figure 4:**
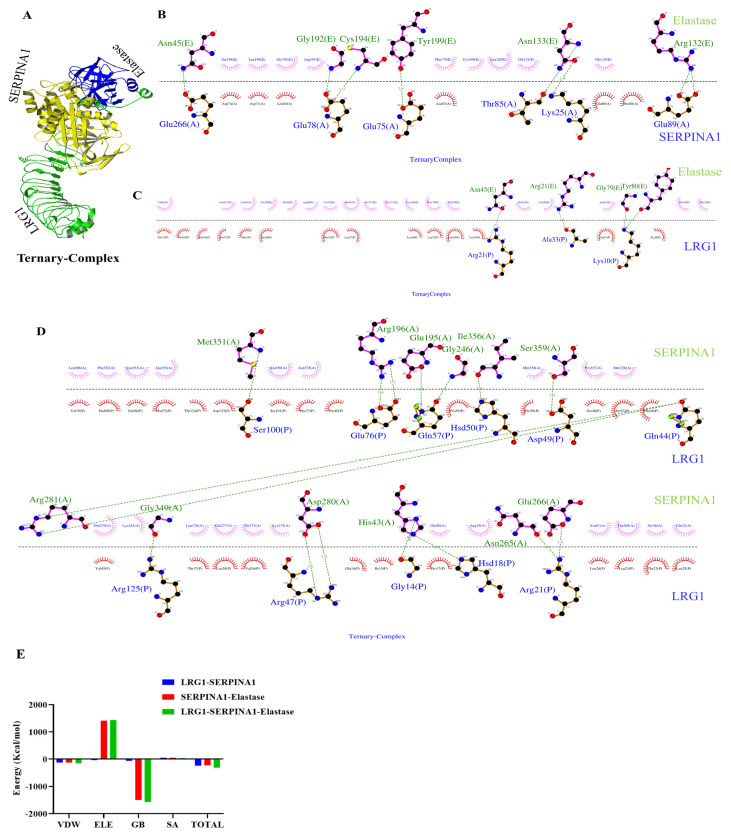
Molecular docking analysis of the Ternary complex and MMGBSA. LRG1–SERPINA1–Elastase docked Ternary complex structure (**A**). LRG1–SERPINA1 complex forms 16 hydrogen bonds (**D**) while SERPINA1–Elastase and LRG1-Elastase form only 8 and 4 hydrogen bonds (**B**,**C**), respectively. Total MMGBSA energy of LRG1–SERPINA1 is greater (−237.82) than that of the SERPINA1–Elastase (−231.76) complex (**E**).

The protein structures of LRG1, SERPINA1, SERPINA3, CLU, and Elastase were retrieved from the RCSB-PDB database with PDB IDs 8H24, 3NE4, 3DLW, 7ZET, and 8VK5, respectively. The active pockets were visualized using Cast-fold (Figure 3A,B). Among the clusters, the molecular docking study revealed that LRG1 binds with SERPINA1, SERPINA3, and CLU with binding energies −1037.5, −1033.8, and −986.2 (kcal/mol), respectively (Supplementary Tables S10–S12). LRG1 showed maximum affinity towards SERPINA1. Since LRG1 showed greater binding energy with both SERPINA1 and SERPINA3, the binding efficiency was further analyzed by comparing the hydrogen bond formation of LRG1 with the active residues of SERPINA1 and SERPINA3. SERPINA1 has Methionine 358 (M358) and Serine 359 (S359) in its reaction center loop (RCL), both of which are active residues [37]. SERPINA3 has active residues located between the amino acid residues 358 and 360 in its RCL [37], important for its protease inhibitor activity. LRG1 binds with the S359 active residue of SERPINA1 with a total of 17 hydrogen bonds (Figure 3C,D). LRG1 also binds with Leucine359 active residue of SERPINA3 with a total of 3 hydrogen bonds (Supplementary Figure S1). The high binding energy of LRG1 with SERPINA1, and a higher number of hydrogen bond formations compared to SERPINA3, showed an efficient interaction between LRG1 and SERPINA1. Our findings thus enable us to further analyze the affinity of the LRG1–SERPINA1 interaction. We have compared the interaction between LRG1 and SERPINA1 with that of SERPINA1’s well-known Interacting partner, Elastase. Elastase is a pro-inflammatory molecule that is highly active in RA [38]. The LRG1–SERPINA1 complex showed higher binding energy (−1037.5 kcal/mol) than the SERPINA1–Elastase complex (−775.7 kcal/mol) (Supplementary Tables S10 and S13). Besides this, the LRG1–SERPINA1 complex showed a higher number of hydrogen bonds than the SERPINA1–Elastase complex, with a total of 17 and 13 hydrogen bonds formed in each complex, respectively (Figure 3D,F). Upon comparing the LRG1–SERPINA1 complex with the SERPINA1–Elastase complex, Elastase was found to bind the active residue Ser359 of SERPINA1. These findings showed LRG1’s possible inhibitory effect on SERPINA1 function. Besides this, Arg196, Arg281, Asp280, and Ser359 are the common residues of SERPINA1 that form hydrogen bonds with both Elastase and LRG1, further showing LRG1’s binding affinity towards SERPINA1. The high binding energy of LRG1, along with a greater number of hydrogen bonds formed with the essential residues of SERPINA1 in the LRG1–SERPINA1 complex compared to SERPINA1–Elastase complex, further showed LRG1–SERPINA1’s binding affinity (Figure 3E,F). We then compared the overall interaction between LRG1, SERPINA1, and Elastase. Ternary complex docking was performed to compare the hydrogen bond formation pattern between the complexes (Figure 4A). The LRG1–SERPINA1 complex revealed 16 hydrogen bonds (Figure 4D), while SERPINA1–Elastase and LRG1-Elastase formed only 8 and 4 hydrogen bonds, respectively (Figure 4B,C). The higher number of hydrogen bonds between LRG1 and SERPINA1 in the ternary complex, along with the greater total MMGBSA energy of LRG1-SERPINA1 (−237.82) compared to SERPINA1-Elastase (−231.76) complex (Figure 4E), further showed LRG1’s binding affinity towards SERPINA1.

### 3.4. MD-Simulation

**Figure 5:**
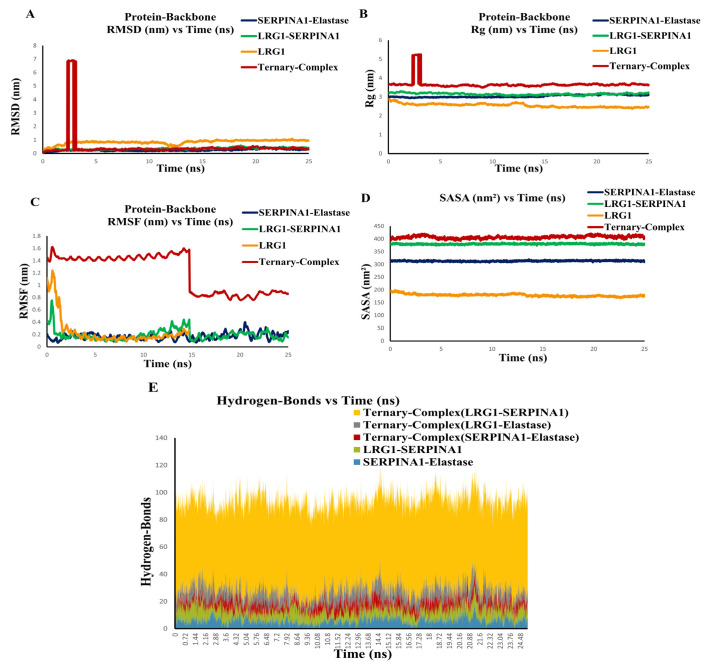
MD simulation of protein–protein complexes. MD simulations of the LRG1–SERPINA1, SERPINA1–Elastase, and ternary docked complex (LRG1–SERPINA1–Elastase) for a 25 ns simulation showing stabilized RMSD after 5 ns, indicating stable binding for both LRG1–SERPINA1 and SERPINA1–Elastase (**A**). The Rg analysis shows that the LRG1–SERPINA1 complex is both compact and stable, surpassing the ternary complex in performance. After 5 ns of simulation, its stability is closely comparable to that of the SERPINA1–Elastase complex, highlighting its effective binding characteristics (**B**). RMSF profiles indicate lower fluctuations in LRG1–SERPINA1 compared to SERPINA1–Elastase, reflecting increased residue-level stability (**C**). LRG1–SERPINA1 has a higher SASA than SERPINA1–Elastase, and both complexes exhibit less fluctuation than the ternary complex, suggesting greater stability (**D**). LRG1–SERPINA1 shows more hydrogen bonds than SERPINA1–Elastase, indicating stronger interactions (**E**).

To explore the dynamic behavior and stability of the complexes, MD simulations were performed [39]. We performed MD simulations of LRG1–SERPINA1, SERPINA1–Elastase, and the ternary complex (LRG1–SERPINA1–Elastase). After a 5 ns MD simulation, the RMSD profiles, which indicate total structural drift, and the Rg profiles, which indicate total compactness, were found to be stabilized (Figure 5A,B). Both RMSD and Rg of the LRG1–SERPINA1 complex exhibited stabilization similar to that of the known interacting partner SERPINA1–Elastase complex, which was observed to have stable binding (Figure 5A,B), respectively. Both LRG1–SERPINA1 and SERPINA1–Elastase complexes displayed greater compactness compared to the ternary complex, indicating enhanced stability of both LRG1–SERPINA1 and SERPINA1–Elastase complexes (Figure 5B). The RMSF profiles reflect residue-level stability and showed less fluctuation in the LRG1–SERPINA1 complex than the SERPINA1–Elastase complex after 15 ns of simulation (Figure 5C). This showed greater residue-level stability in the LRG1–SERPINA1 complex.

Surface area availability is typically analyzed by measuring SASA. Higher SASA indicates a greater surface area exposed to the surroundings, and hence the molecules are less stable. It depends on the overall size of the complex. Generally, larger complexes exhibit higher SASA values than smaller ones. LRG1 (50 kDa)-SERPINA1 (47 kDa) complex has more surface area than SERPINA1(47 kDa)-Elastase (30 kDa) complex as expected. Accordingly, we analyzed SASA fluctuations throughout the 25 ns simulation. Both the LRG1–SERPINA1 and SERPINA1–Elastase complexes demonstrated increased stability and reduced structural fluctuations (Figure 5D). The ternary complex showed lower stability and a larger surface area, as it showed higher SASA fluctuations (Figure 5D). The hydrogen bond profiles depicted the intermolecular interactions that stabilize the complexes. The higher number of hydrogen bonds in the LRG1–SERPINA1 complex compared to the SERPINA1–Elastase complex further showed the stable interaction of the LRG1–SERPINA1 complex (Figure 5E). Further, within the ternary complex, LRG1–SERPINA1 contributed more to the hydrogen bond formation than the SERPINA1–Elastase and LRG1-Elastase complexes (Figure 5E). Therefore, from the analysis of RMSD, Rg, RMSF, SASA, and Hydrogen bond, we may conclude that LRG1 can bind to SERPINA1 and possibly hinder Elastase binding.

## 4. Discussion

RA is a long-lasting disease, an autoimmune disorder that causes ongoing inflammation in the joints. In RA, the RA-FLS are significantly affected, contributing to prominent inflammation, leading to joint swelling and pain [1]. Additionally, both inflammation and fibrosis are well-recognized features of RA-FLS, further complicating the condition and affecting joint function [3,10]. Differentially expressed proteins in the inflammatory and fibrotic responses are crucial for a better understanding of disease pathogenesis [40]. The differentially expressed proteins in RA plasma were previously identified using SWATH, analyzed for their involvement in fibrosis, and found to include 14 genes that exhibit both inflammatory and fibrotic characteristics. Among the 14 genes, the protein cluster showed similar variable scores (FC, GDA score, and Relevance score) with no negative correlations and was selected for further analysis. We understood that, since RA is an inflammatory disease, among the clusters, SERPINA1, SERPINA3, and CLU exhibit anti-inflammatory activity [31,32,33], whereas LRG1 exhibits pro-inflammatory activity. Therefore, studying the pro-inflammatory and anti-inflammatory protein networks could help in elucidating their possible mechanisms of action under inflammatory conditions. In addition, the literature mining showed that LRG1’s high expression in RA plasma is positively correlated with the disease activity score 28 (DAS-28), C-reactive protein (CRP), and erythrocyte sedimentation rate (ESR) [41], strongly suggesting its pathogenic role in RA. Besides this, LRG1’s inflammatory role in CIA mice has been reported and investigated, where LRG1 promotes inflammation by differentiating Th17 cells through the TGF-β pathway, and LRG1’s knockout has been shown to ameliorate arthritis in mice [20]. This strongly suggests LRG1’s potential as a therapeutic target in RA pathogenesis. However, LRG1’s pathogenic activity with respect to RA-FLS has not been investigated in great detail so far, and its fibrotic role remains unexplored. LRG1 is also known to promote fibrotic activity in other diseases, including osteoarthritis [34] and renal fibrosis [35]. Additionally, LRG1 is a strong promoter of the TGFβ pathway [42]. The TGFβ pathway is also associated with the activation of inflammatory and fibrotic drivers, including the Wnt/β-catenin pathway, in human fibroblasts [43]. This information laid the foundation for subsequent studies between the pro-inflammatory LRG1 and anti-inflammatory proteins (SERPINA1, SERPINA3, and CLU). Within the cluster, the high co-expression score between LRG1 and SERPINA1, coupled with increased binding energy and a greater number of hydrogen bonds, suggested a higher binding affinity of the LRG1–SERPINA1 complex. In addition to LRG1’s pro-inflammatory nature, SERPINA1 demonstrated a highly effective ability to reduce inflammation by significantly lowering the expression of the Wnt/β-catenin pathway in the joints of arthritic models [31]. Therefore, interaction analysis between the pro-inflammatory LRG1 and the anti-inflammatory SERPINA1 became an interesting area for further investigation, suggesting that the binding affinity should be analyzed further. The binding pattern of LRG1–SERPINA1 needed to be compared with that of a known SERPINA1-interacting partner. The literature mining revealed that Elastase is one of the strongly binding partners of SERPINA1. SERPINA1 exhibits anti-inflammatory activity by inhibiting proteases [37], whereas Elastase promotes the degradation of tissues [38]. The degradation of tissues activates fibroblast growth factors, stimulates the production of ECM, and promotes fibrotic events [44]. As SERPINA1 interacts with Elastase via its RCL, the cleavage in the RCL results in conformational changes in SERPINA1, and the RCL loop inserts into the core of the SERPINA1 molecule. The insertion causes a rotation of the SERPINA1 molecule, resulting in the translocation of Elastase to the opposite pole of SERPINA1, distorting the enzyme’s structure and inhibiting its function [37,45].

The above results, along with literature reports, suggest that a comparative analysis of LRG1–SERPINA1 interaction with that of SERPINA1–Elastase can further evaluate the binding affinity of the LRG1–SERPINA1 complex. Our comparative docking analysis showed that the LRG1–SERPINA1 complex forms a stable binding interaction. Moreover, the increased hydrogen bonding observed between LRG1 and SERPINA1, in comparison to the interactions between LRG1–Elastase and SERPINA1–Elastase within the ternary complex (LRG1–SERPINA1–Elastase), further substantiates the high affinity of the interaction between LRG1 and SERPINA1. Besides this, the higher total energy (MMGBSA) of the LRG1–SERPINA1 complex suggests a stable LRG1–SERPINA1 interaction.

MD simulation is a leading technique in bioinformatics, enabling the characterization of stable binding interactions in protein–protein and protein–ligand complexes [39]. The simulation of the LRG1–SERPINA1 complex showed stable RMSD and Rg values, similar to those of the SERPINA–Elastase complex. The RMSF indicated reduced fluctuations in the LRG1–SERPINA1 complex, suggesting an efficient binding. Findings on RMSD, Rg, RMSF, SASA, and hydrogen bonding demonstrated a strong interaction between LRG1 and SERPINA1, indicating LRG1’s potential role in modulating SERPINA1’s inhibition of elastase. LRG1 may therefore act as a natural inhibitor of SERPINA1, possibly promoting inflammation and fibrotic events by interfering with SERPINA1’s elastase-inhibitory activity.

## 5. Conclusions

This study reveals a novel stable interaction between LRG1 and SERPINA1. LRG1 promotes inflammation via IL6R regulation, and SERPINA1 inhibits inflammation via inhibiting protease activity, which is very much active in RA pathogenesis. The LRG1 may inhibit SERPINA1 protease-inhibitor activity, thereby promoting inflammation and tissue degradation via elastase. The link between LRG1 and the fibrotic gene set, along with its possible inhibitory activity on the elastase inhibitor SERPINA1, suggests a potential fibrotic and inflammatory role of LRG1 in RA pathogenesis. This information suggests thatLRG1 may be considered as a potential therapeutic target for RA-related symptoms.

## 6. Limitations

Although LRG1 and SERPINA1 interact significantly in in silico studies, an in vitro study is needed to further support their interactions. Additionally, future studies will investigate the effect of LRG1 on the fibrotic aspect of RA pathogenesis.

## Data Availability

For all original data and protocol, please get in touch with Sagarika Biswas (sagarika.biswas@igib.res.in).

## Supplementary Materials

The following supporting information can be downloaded at: https://www.mdpi.com/article/10.3390/pathophysiology33010016/s1, Supplementary Table S1: Upregulated RA plasma proteins, Supplementary Table S2: RA-associated genes from DisGeNET database, Supplementary Table S3: Fibrosis-associated genes from GeneCard database, Supplementary Table S4: Co-expression scores, Supplementary Table S5: Biological Process, Supplementary Table S6: Cellular Component, Supplementary Table S7: Molecular Function, Supplementary Table S8: Reactome Pathway, Supplementary Table S9: Tissue Expression, Supplementary Table S10: LRG1–SERPINA1 docking binding energy, Supplementary Table S11: LRG1-SERPINA3 docking binding energy, Supplementary Table S12: LRG1-CLU docking binding energy, Supplementary Table S13: SERPINA1-Elastase docking binding energy, Supplementary Figure S1: LRG1-SERPINA3 interaction analysis.

## Abbreviations

Alpha Smooth Muscle Actin (αSMA); ExtracellularMatrix (ECM); Fibronectin1 (FN1); Leucine rich Alpha 2 Glycoprotein 1 (LRG1); Molecular Dynamics (MD); Molecular Mechanics Generalized Born Surface Area (MMGBSA); National Center for Biotechnology Information (NCBI); Radius of gyration (Rg); Research Collaboratory for Structural Bioinformatics Protein Data Bank (RCSB PDB); Rheumatoid Arthritis (RA); Rheumatoid Arthritis Fibroblast like Synoviocytes (RAFLS); Root Mean Square Deviation (RMSD); Root Mean Square Fluctuation (RMSF); SERine Protease INhibitor, Clade A, Member 1 (SERPINA1); SERine Protease INhibitor, Clade A, Member 3 (SERPINA3); Solvent Accessible Surface Area (SASA); Transforming Growth Factor Beta (TGF-β).
