# Supplementary material for: Do LRG1–SERPINA1 Interactions Modulate Fibrotic and Inflammatory Signatures in Rheumatoid Arthritis? A Proteomic and In Silico Investigation
Source: Pathophysiology. 2026 Feb 6;33(1):16. doi: 10.3390/pathophysiology33010016 (PMC12922013; doi:10.3390/pathophysiology33010016)
Supplement: Supplementary file 1 [file pathophysiology-33-00016-s001.zip › Supplimentary figure S1.docx]

**Do LRG1-SERPINA1 Interactions Modulate Fibrotic and Inflammatory Signatures in Rheumatoid Arthritis? A Proteomic and In-Silico Investigation.**

Talib Hussain^1,2^, Monika Verma^1,^ Sagarika Biswas^1,2*^

^1^ Integrative and Functional Biology Department, Council of Scientific & Industrial Research (CSIR)-Institute of Genomics and Integrative Biology, Mall Road, Delhi University Campus, Delhi, 110007, India.

^2^ Academy of Scientific and Innovative Research (AcSIR), Ghaziabad, 201002, India.

***Corresponding author**

Dr Sagarika Biswas, PhD.

Chief Scientist

Department of Integrative and Functional Biology

CSIR- Institute of Genomics & Integrative Biology,

Mall Road, Delhi-110 007, India

Tel: +91 11 27667602

& Fax #: +91-11-27667471, #9818004740

E-mail: sagarika.biswas@igib.res.in

**Supplimentary Figure:**

**Supplimentary Figure S1: LRG1-SERPINA3 interaction analysis. 1A)** LRG1-SERPINA3 interaction complex. **B)** Hydrogen bond analysis showing 3 hydrogen formation between LRG1-SERPINA3 complex.


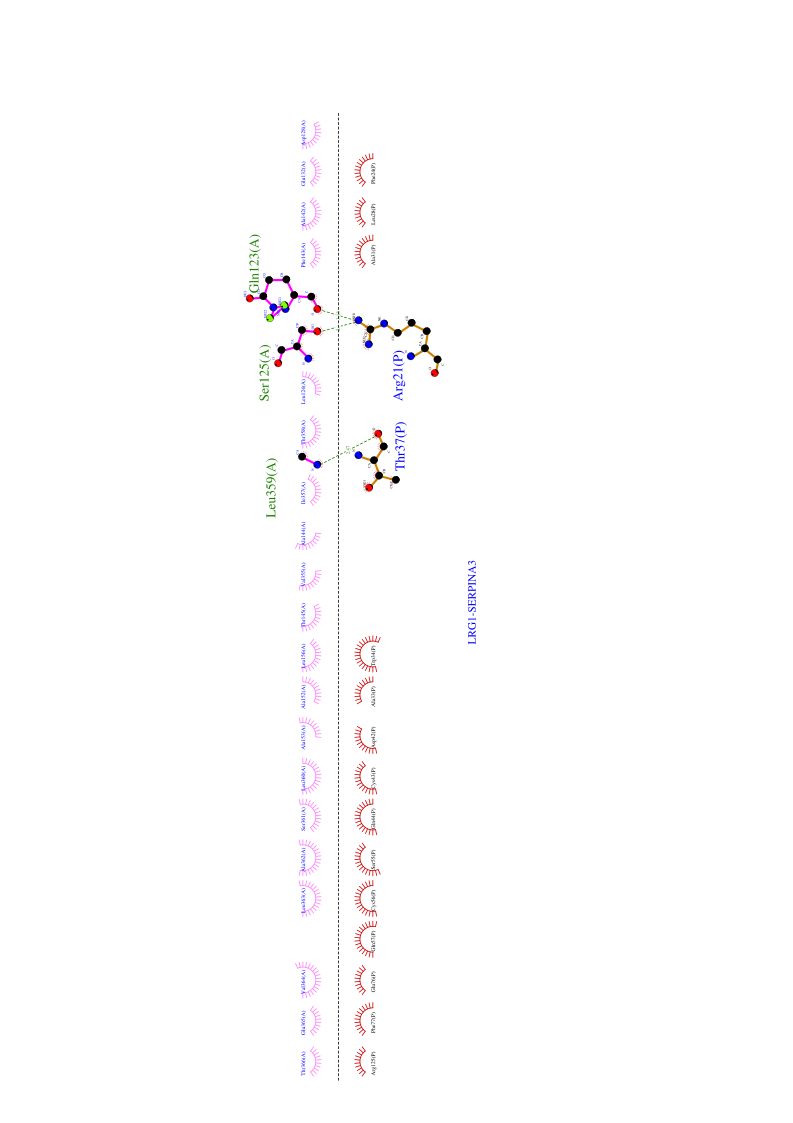

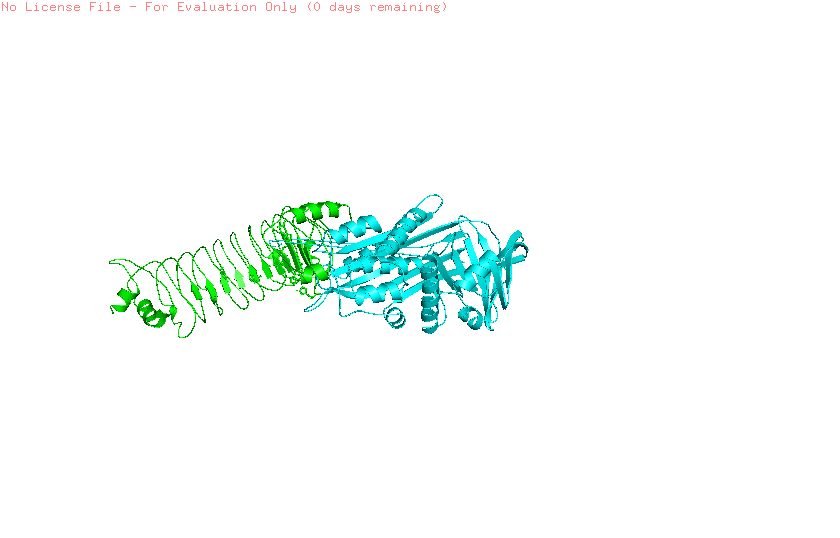


**1B**

**1A**
